# Machine learning-driven assessment of biochemical qualities in tomato and mandarin using RGB and hyperspectral sensors as nondestructive technologies

**DOI:** 10.1371/journal.pone.0308826

**Published:** 2024-08-26

**Authors:** Adel H. Elmetwalli, Asaad Derbala, Ibtisam Mohammed Alsudays, Eman A. Al-Shahari, Mahmoud Elhosary, Salah Elsayed, Laila A. Al-Shuraym, Farahat S. Moghanm, Osama Elsherbiny

**Affiliations:** 1 Agricultural Engineering Department, Faculty of Agriculture, Tanta University, Tanta, Egypt; 2 Department of Biology, College of Science, Qassim University, Unaizah, Saudi Arabia; 3 Department of Biology, Faculty of Science and Arts, King Khalid University, Abha, Saudi Arabia; 4 Evaluation of Natural Resources Department, Agricultural Engineering, Environmental Studies and Research Institute, University of Sadat City, Minufia, Egypt; 5 New Era and Development in Civil Engineering Research Group, Scientific Research Center, Al-Ayen University, Nasiriyah, Iraq; 6 Biology Department, Faculty of Science, Princess Nourah Bint Abdulrahman University, Riyadh, Saudi Arabia; 7 Soil and Water Department, Faculty of Agriculture, Kafrelsheikh University, Kafr El-Sheikh, Egypt; 8 Department of Agricultural Engineering, Faculty of Agriculture, Mansoura University, Mansoura, Egypt; Bahauddin Zakariya University, PAKISTAN

## Abstract

Estimation of fruit quality parameters are usually based on destructive techniques which are tedious, costly and unreliable when dealing with huge amounts of fruits. Alternatively, non–destructive techniques such as image processing and spectral reflectance would be useful in rapid detection of fruit quality parameters. This research study aimed to assess the potential of image processing, spectral reflectance indices (SRIs), and machine learning models such as decision tree (DT) and random forest (RF) to qualitatively estimate characteristics of mandarin and tomato fruits at different ripening stages. Quality parameters such as chlorophyll a (Chl a), chlorophyll b (Chl b), total soluble solids (TSS), titratable acidity (TA), TSS/TA, carotenoids (car), lycopene and firmness were measured. The results showed that Red-Blue-Green (RGB) indices and newly developed SRIs demonstrated high efficiency for quantifying different fruit properties. For example, the R^2^ of the relationships between all RGB indices (RGBI) and measured parameters varied between 0.62 and 0.96 for mandarin and varied between 0.29 and 0.90 for tomato. The RGBI such as visible atmospheric resistant index (VARI) and normalized red (Rn) presented the highest R^2^ = 0.96 with car of mandarin fruits. While excess red vegetation index (ExR) presented the highest R^2^ = 0.84 with car of tomato fruits. The SRIs such as RSI _710_,_600_, and R_730_,_650_ showed the greatest R^2^ values with respect to Chl a (R^2^ = 0.80) for mandarin fruits while the GI had the greatest R^2^ with Chl a (R^2^ = 0.68) for tomato fruits. Combining RGB and SRIs with DT and RF models would be a robust strategy for estimating eight observed variables associated with reasonable accuracy. Regarding mandarin fruits, in the task of predicting Chl a, the DT-2HV model delivered exceptional results, registering an R^2^ of 0.993 with an RMSE of 0.149 for the training set, and an R^2^ of 0.991 with an RMSE of 0.114 for the validation set. As well as for tomato fruits, the DT-5HV model demonstrated exemplary performance in the Chl a prediction, achieving an R^2^ of 0.905 and an RMSE of 0.077 for the training dataset, and an R^2^ of 0.785 with an RMSE of 0.077 for the validation dataset. The overall outcomes showed that the RGB, newly SRIs as well as DT and RF based RGBI, and SRIs could be used to evaluate the measured parameters of mandarin and tomato fruits.

## 1. Introduction

One of the fastest-growing agribusiness industries in Egypt is the production of fruits and vegetables. Citrus and vegetable crops are well adapted to Egypt’s temperate environment. The main fruit crop grown and produced in Egypt is citrus, which is followed by mangoes, grapes, olives, and bananas. Citrus fruits such as oranges (Citrus Aurantium) and mandarin (Citrus reticulate) are among Egypt top exports as in 2020 it was considered the world’s top exporter of citrus. Additionally, Egypt exported nearly 2 million Mg of citrus [1]. In Egypt, mandarins account for roughly 25% of the total citrus production. The total planted area is about 46036 ha with a total production of 1038753 Mg; an average productivity of 22.6 Mg/ha. Citrus juices extracts contain important antioxidants because they are an important source of phenolic compounds [2]. Ahmed et al. [3] also revealed that citrus juice is a rich source of ascorbic acid, vitamins and antioxidants that are important for our health.

Tomato fruits (*Solanum lycopersicum*) are also one of the most important vegetable crops in Egypt, with 168000 hectares cultivated area and 8 million Mg production in 2021. According to FAO [1], Egypt is ranked sixth for exporting tomato fruits with an average exports amount and value of 57.9 thousand Mg and 32.2 million dollars, respectively. Tomato fruits are rich in lycopene, which plays an important role in reducing the incidence of various diseases such as cardiovascular disease, osteoporosis, heart disease and cancer. In addition, tomato fruits are rich in vitamins A and K, vitamin C and potassium [4–6].

Various biochemical properties including total chlorophyll, chlorophyll a and b, carotenoids, soluble solid content (SSC) can effectively be used as fruit quality or maturity/ripening parameters. The determination of fruit quality parameters mainly depends on destructive methods which is difficult to be accomplished when huge numbers of observations are needed. These methods are not reliable for fast changes in fruit quality parameters. According to Wanitchang et al. [7] common destructive methods for measuring fruit ripeness, such as pH, total soluble solids, TSS/TA, and chlorophyll content, result in fruit destruction, take much time, expensive and delay export.

Image processing and spectral reflectance measurements can be robust alternative techniques to conventional methods of assessing fruit quality attributes [8]. Image processing technique can obtain fruit images and acquire their spatial data while spectral reflectance offers data about chemical and physical properties of fruits. Image processing and spectral reflectance can provide the collection of fruit images and spectral data simultaneously [9].

Previous studies investigated the feasibility of spectral reflectance measurements to estimate biochemical properties of fruit as diagnostic indicators of fruit quality. Passive remote detection sensors mainly depend on sunlight as illumination source, which enable hyperspectral data to be collected in the visible (VIS) and near infrared (NIR) regions of the electromagnetic spectrum [9–11]. Elsayed et al. [9] revealed that the newly developed (NDVI-VARI)/(NDVI-VARI) index showed a remarkable correlation with chlorophyll t, chlorophyll a and chlorophyll b of mango with R^2^ values of 0.71, 0.71 and 0.78, respectively. Salah et al. [12] used spectroscopic technique to predict the chemical properties of orange fruits at different growth stages showing that the NDIs and R_672_/R_550_ had strong significant correlations with chlorophyll b and chlorophyll a (R^2^ = 0.84 and 0.92, respectively) while PSI and R_672_/R_550_ had the highest correlation with TSS. Borba et al. [13] quantified the quality properties of tomato fruits in a non-destructive manner using spectral reflectance. They concluded that total soluble solid content (TSS), titrated acidity (TA) and citric acid can be rapidly and efficiently estimated using spectroscopy measurements.

The assessment of fruit quality in crops using SRIs often yields inconsistent outcomes in diverse geographical and environmental conditions. Therefore, there is an ongoing need for refining SRIs and RGBI to enhance their effectiveness as a rapid and straightforward approach for accurately estimating fruit quality parameters. It is of utmost importance to ascertain the optimal algorithmic formulations for the computation of diverse fruit quality attributes, thereby enhancing the efficacy of remotely acquired data in the evaluation of fruit quality. Typically, prior studies have primarily concentrated their efforts on utilizing published SRIs for the assessment of various fruit quality attributes. As far as the authors are aware, only a limited number of inquiries have investigated into the simultaneous application of distinct techniques for fruit property assessment. The distinctive advantage of the present investigation lies in the methodology employed for selecting the most suitable SRIs for the evaluation of fruit quality parameters. In this regard, the utilization of correlogram maps stands out as a noteworthy approach, enhancing the study’s capability to identify and employ the most effective SRIs in assessing fruit quality attributes.

Although SRIs offer a straightforward approach for the estimation of biochemical parameters, with the potential to enable the development of a portable and lightweight instrument for the rapid and cost-effective assessment and management of biochemical parameters on a significant scale, it is important to note that each SRI is constrained by a finite set of band combinations. The challenge lies in formulating robust SRIs for the assessment of fruit quality attributes amidst diverse and potentially perplexing conditions. These conditions encompass substantial variations in the dimensions of fruit components and their consequential impact on the saturation level of the quality parameters under scrutiny. As a subset of artificial intelligence (AI), machine learning (ML) has grown quickly in this environment. A huge amount of spectrum data can be used by ML to extract important information for accurate classification and self-prediction [14]. Employing model-based techniques for the purpose of feature selection has the capacity to discern a subset of features characterized by substantial discriminative and predictive potential, as demonstrated by the research conducted by Beltrán et al. [15]. This strategy has the potential to augment model performance by mitigating the issue of overfitting and removing extraneous features. Additionally, retaining the initial feature representation can contribute to enhanced interpretability, as highlighted by Guyon and Elisseeff [16]. The significance of feature selection algorithms in the context of modeling and prediction has been steadily on the rise, as underscored by Schuize et al. [17]. Numerous approaches have been investigated for the purpose of diminishing data dimensionality. These include Decision Trees (DT) and Random Forest (RF). In the RF model, an assessment of variable importance is carried out based on the methodology outlined by Strobl et al. in their seminal work [18]. Glorfeld et al. (2019) introduced a back-propagation neural network metric aimed at discerning the most pivotal variables within a given context [19]. Furthermore, the process of hyper-parameter selection wields substantial influence over the performance of ML models, yielding manifold advantages. For instance, it has the potential to augment the efficacy of ML algorithms [20], foster equity and replicability in the realm of scientific inquiries [21], and exert a direct influence on the training dynamics of algorithms, thereby assuming a pivotal role in the enhancement of predictive models [22].

In the context of this research study, the overarching objective was to assess the effectiveness of both RGBI and SRIs as non-destructive techniques for estimating the characteristics of mandarin and tomato fruits, as well as for detecting the quality parameters of these fruits at various stages of maturity. To achieve this, the study set out to accomplish the following specific goals: (i) Quantify the quality parameters of mandarin and tomato fruits at different stages of ripening; (ii) Evaluate the suitability of both conventional and newly developed SRIs for quantifying the quality parameters of mandarin and tomato fruits; and (iv) Assess the performance of DT and RF models, which are based on RGBI and SRIs, in predicting the quality parameters of mandarin and tomato fruits.

## 2. Materials and methods

### 2.1. Plant material

**Fig 1:**
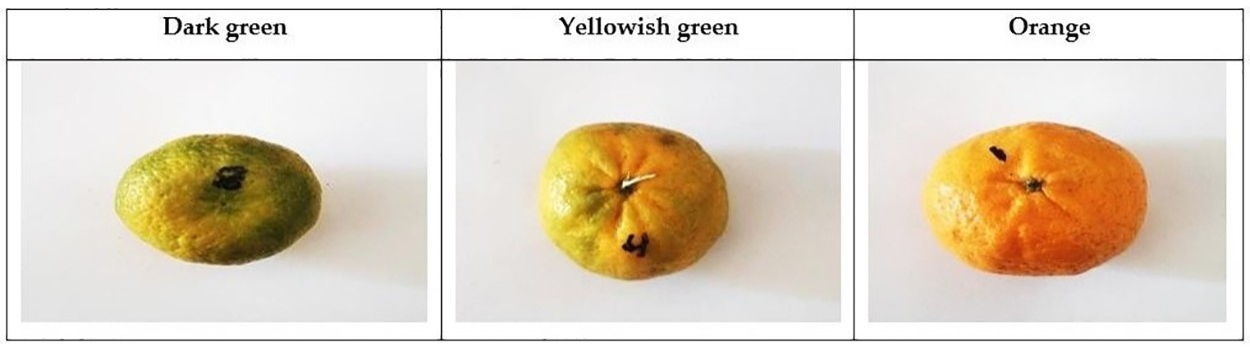
Mandarin images at different ripening stages.

**Fig 2:**
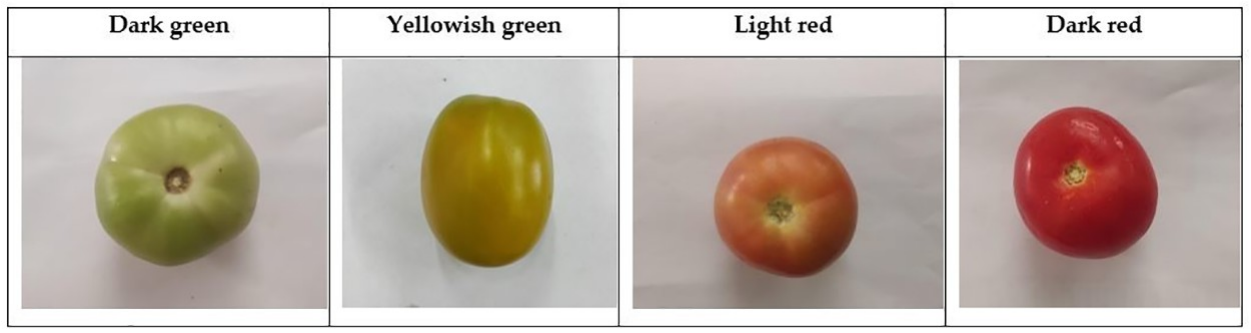
Tomato images at different ripening stages.

The experiments were conducted on mandarin and tomato fruits in the Laboratory of the Faculty of Agriculture, Tanta University, Gharbia Governorate, Egypt (30° 47’ 18.00"N and 30° 59’ 54.61"E). Samples of fruits were collected from a private farm in Gharbia Governorate, a different stages of ripening. The fruits were randomly selected and harvested manually. The experiments were conducted throughout the year 2021 to predict the quality attributes of mandarin and tomato fruits using RGB indices and SRIs and linking them to the biochemical properties of the fruits. Balady Mandarin (*Citrus reticulata*, Blanco) fruit specimens of the seven-year-old trees were procured during three distinct phases of ripening: the mature stage, characterized by predominantly green coloration; the semi-ripening stage, exhibiting a combination of green and yellow hues; and the ripening stage, marked by a vivid orange hue, as visually represented in Fig 1. Tomato fruits (*Solanum lycopersicum*, Alissa F1) were collected at four different ripening such as dark green, yellowish green, light red and dark red as shown in Fig 2 and they were used for laboratory analysis.

### 2.2. Chemical parameters

#### 2.2.1. Chlorophyll a, Chlorophyll b, carotenoids and lycopene

A spectrophotometer was used to measure the absorbance at certain wavelengths of 663, 645, 480 and 503 nm to determine the content of Chl a, Chl b, car [23] and lycopene [24] of crude extracts in mandarin and tomato fruits using the following equations:

$$\text{Chlorophyll}\;\text{a}\;\text{mg}/\text{g}\;\text{tissue}=12.7({\text{A}}_{663})-2.69(A_{645})\times \frac{V}{1,000\times W}$$ (1)

$$\text{Chlorophyll}\;\text{b}\;\text{mg}/\text{g}\;\text{tissue}=22.9({\text{A}}_{645})-4.68({\text{A}}_{663})\times \frac{V}{1,000\times W}$$ (2)

$$\text{Total}\;\text{carotenoids}\;(\text{mg}/\text{g}\;\text{fw})=[A_{480}+(0.114\times A_{663})-(0.638-{\text{A}}_{645})]\times \frac{V}{1,000}\times W$$ (3)

$$\text{Lycopene}\;\;(\mu \text{g}/\text{g}\;\text{fr}.\text{wt}.)=\frac{3.121\times OD\;value\;at\;503\;nm\times vol.\;of\;sample\times Dilution\;factor}{Fresh\;weight\;of\;sample\;(g)}$$ (4)

Where A = absorbance at specific wavelengths, W = fresh weigh, and V = final volume of chlorophyll extract.

#### 2.2.2. Total soluble solids (TSS)

A handheld refractometer (Milwaukee, model MA871, Brookfield, WI, USA) was used to measure the TSS in juice extract from tomato and mandarin fruits and the data was expressed as Brix (%) according to Cheour et al. [25].

#### 2.2.3. Titrated acidity (TA)

The titrated acidity of tomato and mandarin juice extracts as a percentage of anhydrous citric acid was measured by titrating a given volume of juice fruits known to 0.1 N NaOH standard using 1% phenolphthalein as an indicator by A.O.A.C. [26].

#### 2.2.4. Maturity index (TSS/TA)

The TSS/TA ratio was calculated from the TSS values divided by the TA values.

### 2.3. Physical parameter

The firmness of each fruit mandarin and tomato was measured using a digital fruit hartester (IC-FR5120, China) with a 6 mm probe.

### 2.4. Image analysis

Environment for Visualization and Visualization 4.6 (ENVI 4.6) is the ideal software (ITT, Visual Information Solutions, Boulder, CO, USA) for visualizing, viewing and analyzing digital images of all types. In addition, a large number of ENVI wizards are available, covering almost all functions available in the interactive ENVI software. Each processing routine is an IDL operation or function and is used like any other IDL routine. The image analysis results take the average value of three different RGB bands. The fruits of the mandarin and tomato were photographed with a Nikon D5300 camera (Nikon Corporation, Tokyo, Japan), a 24.2 megapixels digital single-lens reflex (DSLR) camera with 18–55 mm lens. The camera was manually held and directed vertically downwards towards the mandarin and tomato fruits at a distance of 30 cm. The measurements were carried out under cloudy conditions to guarantee high image resolution. The flash of the camera was always kept off during measurements using IrfanView 4.37, the digital photographs were converted from JPEG to TIF file format. Statistical analysis was done using the SPSS 22 package to calculate selected RGBI based on the red (R), green (G), and blue (B) pixels (Table 1).

**Table 1:** Index abbreviations, formulae, and references of selected RGB indices for digital image analysis.

Index Abbreviation	Formulae	Reference
Nomalized red (Rn)	G/(R + G + B)	[27]
Nomalized blue (Bn)	B/(R + G + B)	[27]
Kawashima index (IKAW)	(R−B)/(R+B)	[9]
Excess red vegetation index (EXR)	1.4×rn−gn	[28]
Excess green minus excess red index (ExGR)	ExG–ExR	[29]
Color index of vegetation (CIVE)	0.441×R– 0.881×G+0.385×B+18.78745	This study
Green red ratio index (GRRI)	G/R	[30]
Green-red vegetation index (GRVI)	(G−R)/(G+R)	This study
Normalized difference index (NDI)	(rn−gn)/(rn+gn+0.01)	[12]
Visible Atmospheric Resistant Index VARI	$\frac{\left(\text{Green}-\text{Red}\right)}{\left(\text{Green}+\text{Red}-\text{Blue}\right)}$	[31]
VARI1	$\frac{\left(\text{Green}-\text{VARI}\right)}{\left(\text{Green}+\text{VARI}+\text{Blue}\right)}$	[9]
Normalized Difference Vegetation Index 1 (NDVI1)	$\frac{\left(\text{Red}-\text{Blue}\right)}{\left(\text{Red}+\text{Blue}\right)}$	[32]
Normalized Difference Vegetation Index (NDVI)	$\frac{\left(\text{Red}-\text{Green}\right)}{\left(\text{Red}+\text{Green}\right)}$	[33]
Color intensity index (INT)	(R+G+B)/3	[34]

### 2.5. Spectral reflectance measurements

Following the acquisition of various samples of mandarin and tomato, representing distinct ripening stages, spectral data for each specimen were obtained utilizing a passive reflection sensor manufactured by HandySpec Field^®^ (tec5, Oberursel, Germany). The spectral range encompassed wavelengths spanning from 302 to 1148 nanometers (nm). Notably, the optical bandwidth and perspective angle employed in this spectral data acquisition process were set at 2 nm and 12 degrees, respectively. Each sample was scanned three times. To avoid exposure differences, the spectroradiometric measurements of the different samples were performed in full sunlight for short periods of time. Spectral reflectance matching of different samples was done using calibration factors derived from a white reference standard. In the course of spectral measurements, it was imperative to employ a black sheet positioned beneath the fruit specimen. This strategic placement served the crucial purpose of mitigating spectral reflections emanating from the surrounding background, thereby ensuring that the recorded spectral data primarily represented the reflective characteristics of the fruit itself. From the readings of the spectrometer unit, the reflectance of the fruit is calculated and corrected using calibration elements taken from the reference gray standard. Spectroradiometric measurements were taken from the vertical position approximately 30 cm above the fruit on clear days.

#### 2.5.1. Selection of SRIs of tomato and mandarin fruits

**Fig 3:**
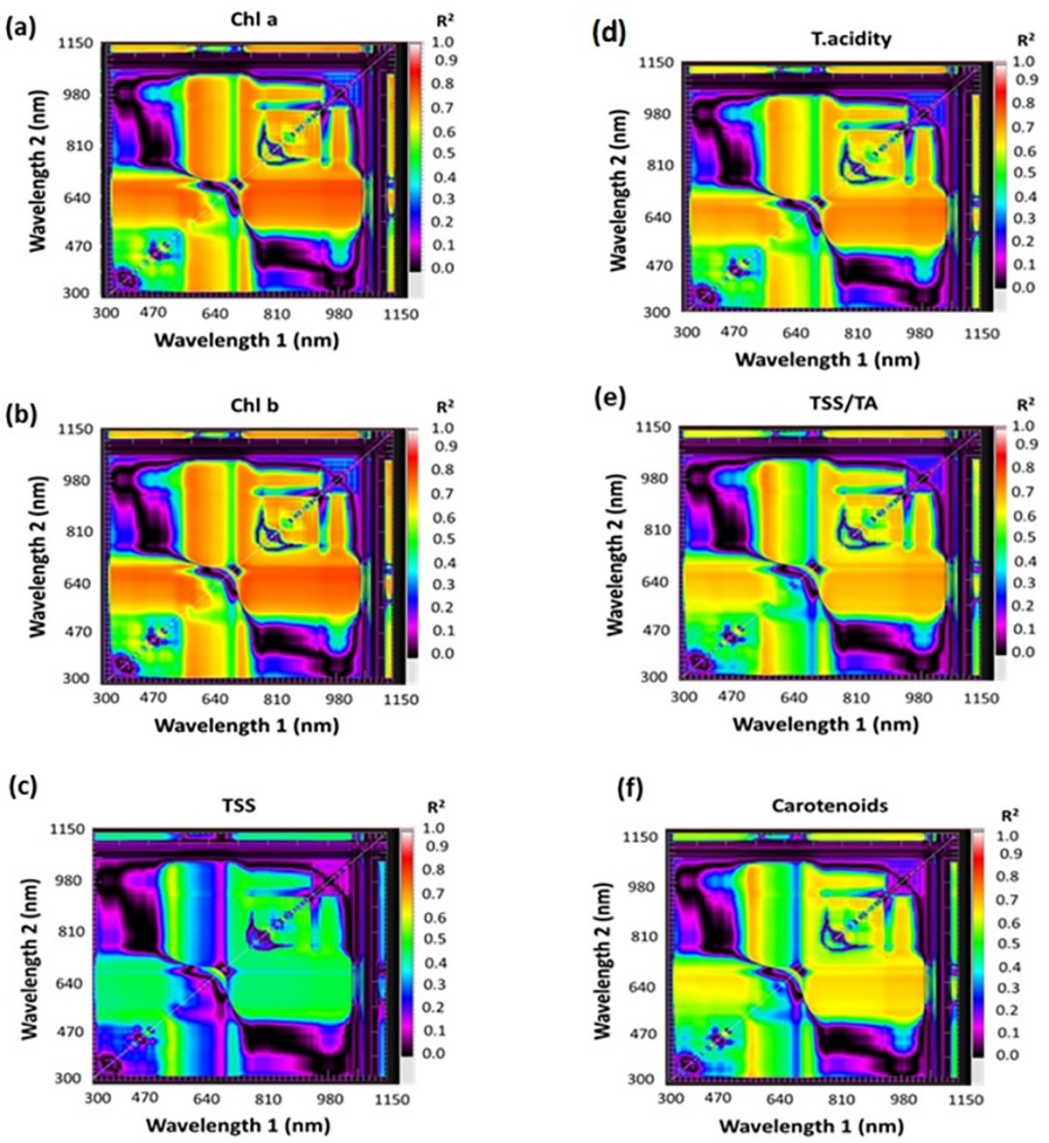
Contour maps (Correlation matrices) illustrating the R^2^ for two sets wavelength pairings in the 302–1148 nm range (as a ratio index) with (a) chlorophyll a (Chl a), (b) chlorophyll b (Chl b), (c) total soluble solids (TSS), (d) titratable acidity (TA), (e) TSS/TA, and (f) carotenoids of mandarin fruits at different ripening degrees.

**Fig 4:**
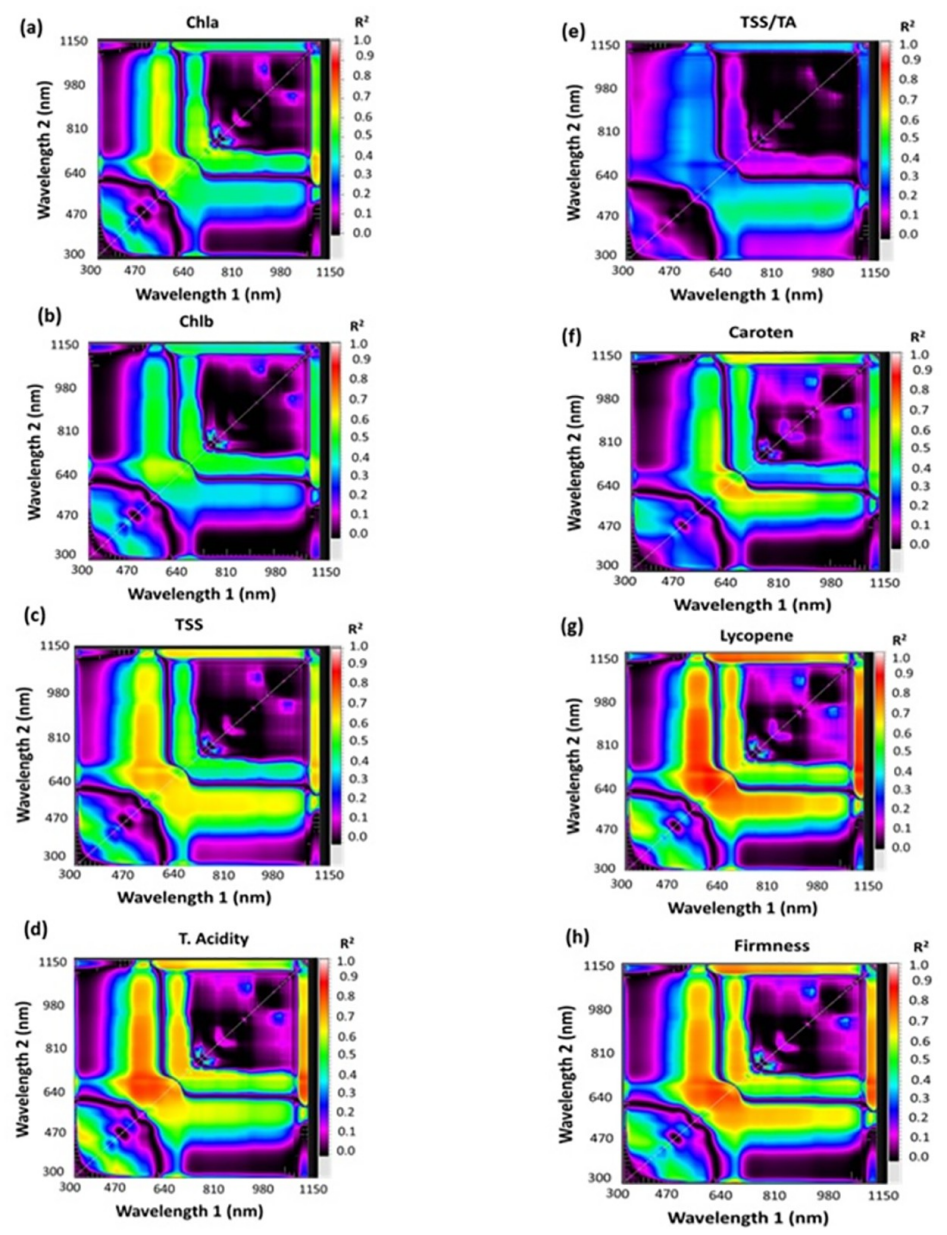
Contour maps (Correlation matrices) illustrating the R^2^ for two sets wavelength pairings in the 302–1148 nm range (as a ratio index) with (a) chlorophyll a (Chl a), (b) chlorophyll b (Chl b), (c) total soluble solids (TSS), (d) titratable acidity (TA), (e)TSS/TA, (f) carotenoids (car), (g) lycopene and (h) firmness of tomato fruits at different ripening degrees.

Table 2 contains a list of selected SRIs, including both published and newly generated indices. Contour maps of correlation matrices exhibited statistical metrics in the form of determination coefficients (R^2^) among the measured values of mandarin and tomato fruits with ratio spectral indices (RSI) as seen in Figs 3 & 4. The RSI was calculated by merging a pair of wavelengths in the 302–1148 nm spectrum range (Figs 3 and 4). Elsayed et al. [35], developed spectral contour maps to determine the most efficient spectrum region with productive wavelengths and to identify the significance of SRIs. SRIs were computed using several wavelengths (522, 534, 546, 550, 566, 584, 600, 608, 610, 616, 618, 620, 622, 632, 640, 646, 648, 650, 654, 660, 664, 666, 670, 672, 674, 676, 678, 710, 720, 730, 750, 760, 780, 810, 822, 824, 878, 894, 1120, 1132 and 1140).

**Table 2:** Index abbreviations, formulae, and references of new and published spectral indices used in the present study.

SRIs	Formulae	Reference
Ratio Spectral Indices (RSI)		
RSI _710,600_	R_710_/R_600_	This work
RSI _730,650_	R_730_/R_650_	
RSI _676,664_	R_676_/R_664_	
RSI _672,670_	R_672_/R_670_	
RSI _678,666_	R_678_/R_666_	
RSI _894,878_	R_894_/R_878_	
RSI _824,810_	R_824_/R_810_	
RSI _810,822_	R_810_/R_822_	
RSI _654,566_	R_654_/R_566_	
RSI _546,1132_	R_546_/R_1132_	
RSI _534,750_	R_534_/R_750_	
RSI _522,750_	R_522_/R_750_	
RSI _632,608_	R_632_/R_608_	
RSI _1140,674_	R_1140_/R_674_	
RSI _616,674_	R_616_/R_674_	
RSI _610,646_	R_610_/R_646_	
RSI _622,640_	R_622_/R_640_	
RSI _648,1120_	R_648_/R_1120_	
RSI _620,618_	R_620_/R_618_	
RSI _640,616_	R_640_/R_616_	
RSI _584,650_	R_584_/R_650_	
RSI _610,650_	R_610_/R_650_	
Normalized Difference Vegetation Index (NDVI)	(R_780_-R_660_) / (R_780_ + R_660_)	[36]
Anthocyanin index (NAI)	(R_760_-R_720_) /(R_760_ + R_720_)	[37]
Greenness index (GI)	R_554_/R_677_	[38]
Pigment-sensitive ripening monitoring index (PRMI)	(R_750_-R_678_)/R_550_	[11]

### 2.6. Machine learning models

#### 2.6.1. Random Forest (RF)

**Fig 5:**
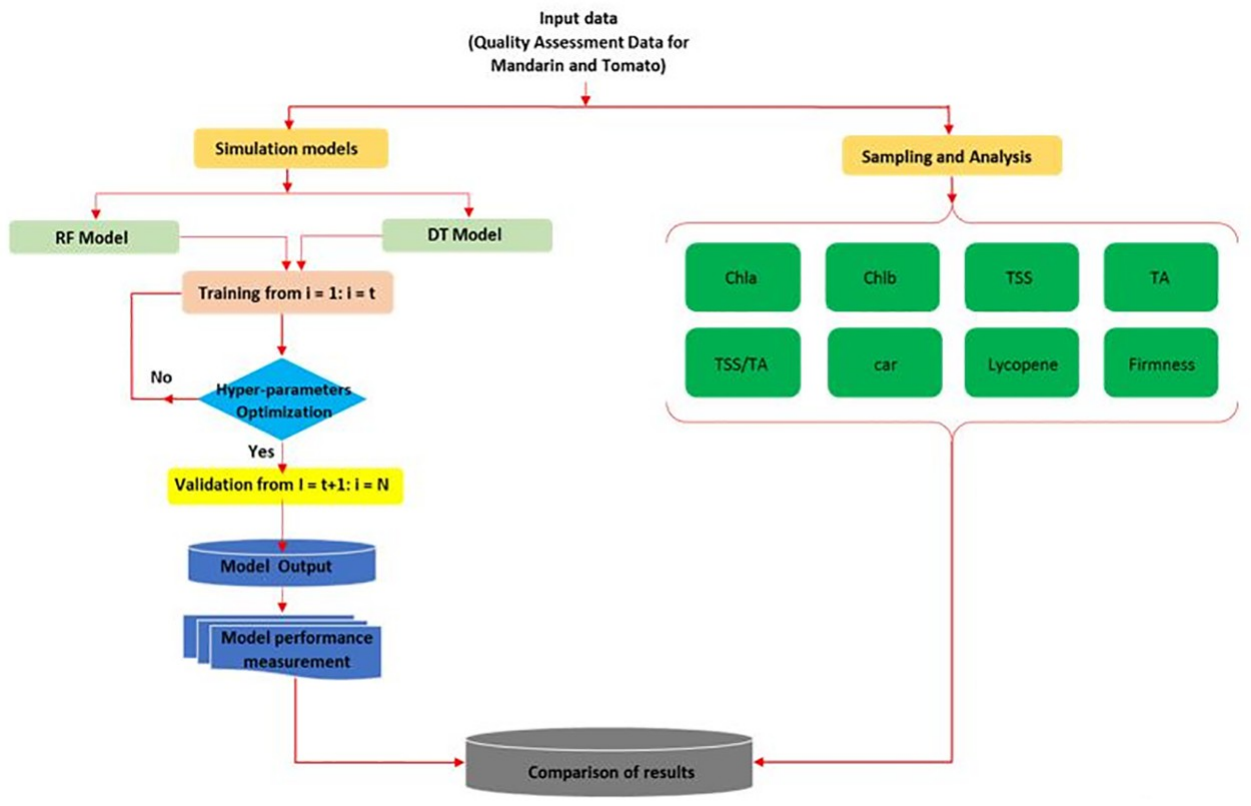
Flowchart for predicting eight fruit parameters of mandarin and tomato using spectral and RGB indices with decision tree (DT) and random forest (RF) models.

Random forest (RF) is a versatile technique grounded in regression trees or multiple classifications, adept at assessing the interplay among a number of variables that are independent and dependent variables. It achieves this by partitioning the dataset into various nodes, forming homogeneous subsets known as regression trees (ntree) through recursive partitioning, and subsequently aggregating the outcomes from all these trees. In its growth phase, each tree is expanded to its greatest extent, drawing upon a bootstrap sample from the dataset used for training, and notably, it introduces an element of randomness during the regression step within each tree. This randomness is introduced by selecting a random subset of variables (mtry) to estimate the node split at each juncture [18]. The training process of RF takes into consideration three critical factors: ntree and mtry. Specifically, ntree is the number of trained features, ranging from 1 to 20, while mtry corresponds to the random subset of features selected for node splitting. To optimize the model and minimize the root mean squared error (RMSE) of validation (RMSEV), the leave-one-out validation method (LOOV) is employed for fine-tuning the two parameters, mtry and ntree. The parameter ntree undergoes scrutiny in the range of 1 to 25, and the optimal value for mtry is assessed by varying the number of features used. Once the model is trained with the optimal parameters (Fig 5), all the features are organized, and a selection of the most valuable features is made based on variable importance statistics [39]. Throughout this iterative process, the outputs are diligently collected, and multiple combinations of features are assessed to identify the optimal feature set that yields the lowest RMSEV.

#### 2.6.2. Decision Tree (DT)

The process known as decision tree induction is the method by which decision trees are generated from sets of training data that have been annotated with class labels. These decision trees take on the form of graphical structures resembling flowcharts, consisting of distinct types of nodes, including a root node, decision nodes, and leaf nodes. The root node marks the starting point of the tree, while the decision nodes play a pivotal role in making choices and guiding the progression from one node to another. Ultimately, the leaf nodes represent the ultimate outcomes or classifications determined by the decision tree. It’s important to note that not all decision tree algorithms produce the same types of trees. Some, such as the CART (Classification and Regression Trees) algorithm, are constrained to produce binary trees, which are characterized by having precisely two internal nodes, while others possess the capacity to generate non-binary trees [40]. During the training phase of decision tree induction, three critical factors are carefully considered: the maximum depth of the tree (Md), the minimum number of samples allowed per leaf (Ms), and the maximum number of leaf nodes (Mln). Specific parameter values have been selected for each of these factors, including Md values of 1, 3, 5, 7, and 9, Ms values of 2, 4, 6, 8, and 10, and Mln values of None, 10, 20, 30, 40, and 50. These parameter choices have a profound impact on the resulting decision tree’s structure, complexity, and its ability to effectively make predictions and generalize in various practical applications. Hyper-parameter optimization (Fig 5) was done during the training phase, resulting in the creation of the top-level model using the most effective parameter settings. Decision tree regressors can be effortlessly derived from decision trees. Due to their minimal reliance on domain expertise or parameter configuration, decision tree regressors are well-suited for exploratory knowledge discovery tasks. Importantly, decision trees exhibit a remarkable ability to accurately handle datasets with high dimensions.

#### 2.6.3. Data analysis tools and datasets

A total of 68 samples each of tomato and mandarin were used for both training and validation purposes. The methodology employed for this task was the leave-one-out cross-validation (LOOCV) technique, which systematically excluded one sample during each trial from the training dataset to facilitate validation. This strategic approach served the dual purpose of mitigating overfitting tendencies and enhancing the model’s predictive capabilities [41]. The entire process, spanning from data analysis to model creation and data preparation, was executed using Python 3.7.3. For the regression tasks, the RF and DT modules from the Scikit-learn package version 0.20.2 were employed. Notably, data examination was performed on a machine equipped with an Intel Core i7-3630QM processor clocked at 2.4 GHz, complemented by 8 GB of RAM.

#### 2.6.4. Model assessment

To evaluate the efficacy of a regression model, two distinct statistical metrics were employed including the coefficient of determination (R^2^) and the root mean square error (RMSE) according to Eqs 5 & 6 [42, 43]. The parameters applied in this assessment are explicitly defined as follows: "Y_act_" signifies the genuine laboratory-derived value, "Y_p_" represents the anticipated or simulated value, "Y_ave_" denotes the mean value, and "T" encompasses the entirety of data points.

Root mean square error

$$RMSE=\sqrt{\frac{1}{N}\sum_{i=1}^{T}{\left(Y_{act}-Y_{p}\right)}^{2}}$$ (5)

Coefficient of determination

$$R^{2}=\frac{\sum {\left(Y_{act}-Y_{p}\right)}^{2}}{\sum {\left(Y_{act}-Y_{ave}\right)}^{2}}$$ (6)

## 3. Results and discussion

### 3.1. Variation of different biochemical parameters, RGB and spectral reflectance indices of mandarin and tomato fruits

Ripening degrees have a significant impact on the biochemical characteristics of mandarin and tomato. The Chl a, Chl b, TA, and firmness values of fruits decreased during the ripening stages. While TSS, TSS/TA, Car, and Lycopene increased with increasing the fruit’s ripening. Significant difference between the mean values of each biochemical parameter at different ripening degrees was found. As well as RGB indices and SRIs values were changed according to the ripening stages in Tables 3 & 4. Chl a values of mandarin ranged from 0.03 to 4.24 (mg/g tissue), and Car from 0.07 to 0.23 mg/g FW, while t RGB indices such as VARI ranged from -0.65 to 0.34, and IKAW from 0.00 to 0.97 (Table 3). In addition, the SRI such as R_710_/R_600_ values ranged from 1.26 to 3.91, and PRMI ranged from 0.28 to 5.78.

**Table 3:** Statistical summary of six biochemical parameters, twelve RGB indices, and twelve spectral reflectance indices for mandarin fruits.

Stage	Unripe			Ripe			Overripe		
Parameters	Min	Max	Mean	Min	Max	Mean	Min	Max	Mean
Chl a (mg/g tissue)	3.56	4.24	3.92a	0.22	0.54	0.39b	0.03	0.16	0.08c
Chl b (mg/g tissue)	4.97	6.23	5.57a	0.36	0.84	0.62b	0.06	0.19	0.11c
TSS (Brix, %)	8.80	10.20	9.56b	10.00	12.00	10.85ab	10.90	12.00	11.70a
TA (%)	1.15	1.42	1.31a	0.68	0.77	0.72b	0.60	0.67	0.64bc
TSS/TA	6.82	8.56	7.33c	13.08	17.41	15.07b	16.81	19.61	18.35a
Carotenoids (mg/g fw)	0.07	0.09	0.08c	0.14	0.21	0.17b	0.21	0.23	0.22a
Rn	0.29	0.36	0.31c	0.42	0.66	0.57b	0.68	0.80	0.73a
Bn	0.14	0.30	0.24a	0.01	0.18	0.04b	0.01	0.04	0.02b
GRRI	1.20	1.66	1.44a	0.46	0.98	0.70b	0.22	0.46	0.34c
GRVI	0.09	0.25	0.18a	-0.37	-0.01	-0.19b	-0.64	-0.37	-0.50c
NDI	-0.25	-0.09	-0.18c	0.01	0.37	0.19b	0.37	0.63	0.49a
IKAW	0.00	0.43	0.15c	0.40	0.97	0.85b	0.89	0.96	0.94a
VARI	0.15	0.34	0.26	-0.38	-0.01	-0.20	-0.65	-0.38	-0.51
ExR	-0.08	0.07	-0.01c	0.18	0.62	0.41b	0.64	0.95	0.78a
ExGR	0.11	0.67	0.36a	-0.71	0.22	-0.25b	-1.41	-0.71	-1.04c
VARI1	0.56	0.78	0.65	0.69b	0.98	0.91a	0.88	0.97	0.93a
NDVI1	0.00	0.43	0.15c	0.40	0.97	0.85b	0.89	0.96	0.94a
NDVI	-0.25	-0.09	-0.18c	0.01	0.37	0.19b	0.37	0.64	0.50a
RSI _710_,_600_	2.67	3.91	3.44a	1.55	1.95	1.82b	1.26	3.19	1.61bc
RSI _730_,_650_	6.39	11.19	9.23a	1.85	2.93	2.57b	1.14	8.35	2.27bc
RSI _676_,_664_	0.95	1.11	1.08a	0.82	1.08	0.96b	0.84	0.87	0.86c
RSI _672_,_670_	1.00	1.02	1.02a	0.97	1.02	0.99ab	0.98	0.98	0.98b
RSI _678_,_666_	1.01	1.15	1.11a	0.92	0.95	0.94b	0.90	1.11	0.93b
RSI _894_,_878_	0.99	1.00	0.99a	0.97	0.99	0.98ab	0.97	0.99	0.98ab
RSI _824_,_810_	1.00	1.01	1.00a	0.96a	0.99	0.95b	0.94	0.98	0.93c
RSI _810_,_822_	0.99	1.00	1.00b	1.01	1.01	1.01a	1.00	1.01	1.01a
RSI _654_/_R566_	0.46	0.65	0.55c	0.72	0.93	0.79b	0.61	1.58	1.22a
NDVI	0.81	0.87	0.84a	0.47	0.66	0.60b	0.08	0.83	0.31c
NAI	0.13	0.27	0.22a	0.02	0.05	0.04b	0.01	0.26	0.03bc
PRMI	2.86	5.78	5.14a	1.22	1.88	1.66b	0.28	5.46	1.27bc

**Table 4:** Maximum, minimum and mean values of eight biochemical parameters of tomato fruits, twelve RGB indices derived from digital image analysis and sixteen spectral reflectance indices.

	Green dark			Yellow green			Red light			Red dark		
Parameters	Min	Max	Mean	Min	Max	Mean	Min	Max	Mean	Min	Max	Mean
Chl a (mg/g tissue)	0.90	1.57	1.30a	0.81	0.98	0.92b	0.61	0.88	0.79c	0.56	0.80	0.70d
Chl b (mg/g tissue)	0.88	1.51	1.26a	0.79	1.20	0.98b	0.56	1.02	0.82c	0.47	0.97	0.70d
TSS (Brix, %)	4.00	5.20	4.58d	4.70	5.60	5.15c	5.30	6.00	5.60b	5.70	6.80	6.00a
TA (%)	0.62	0.71	0.68d	0.74	0.76	0.75c	0.77	0.80	0.79b	0.82	0.83	0.82a
TSS/TA	6.13	7.28	6.69c	6.30	7.39	6.87c	6.73	7.53	7.11b	6.89	8.27	7.28a
Carotenoids (mg/g fw)	0.10	0.13	0.12d	0.11	0.15	0.14c	0.14	0.19	0.16b	0.21	0.29	0.22a
Lycopene (μg/g)	4.53	12.48	9.39d	14.04	42.76	22.75c	34.17	50.09	42.98b	50.87	65.70	60.28a
Firmness (Kg_f_/cm^2)^	610.00	690.00	647.50a	510.00	610.00	548.24b	410.00	510.00	476.47c	350.00	420.00	383.44d
Rn	0.30	0.42	0.36d	0.40	0.57	0.48c	0.50	0.71	0.63b	0.75	0.93	0.84a
GRRI	0.95	1.28	1.16a	0.58	1.09	0.86b	0.36	0.71	0.50c	0.04	0.22	0.13d
INT	132.25	176.31	151.62a	107.81	159.09	132.53b	92.68	136.92	108.13c	68.66	100.88	78.70d
GRVI	-0.03	0.12	0.07a	-0.27	0.04	-0.08b	-0.47	-0.17	-0.34c	-0.92	-0.65	-0.78d
NDI	-0.12	0.03	-0.07d	-0.04	0.27	0.08c	0.17	0.47	0.34b	0.64	0.91	0.77a
IKAW	-0.06	0.65	0.22c	0.26	0.84	0.65b	0.55	0.96	0.81a	0.79	0.97	0.89a
VARI	-0.04	0.21	0.10a	-0.30	0.05	-0.10b	-0.49	-0.20	-0.37c	-0.98	-0.67	-0.82d
ExR	0.04	0.18	0.09d	0.14	0.47	0.27c	0.35	0.74	0.57b	0.89	1.26	1.07a
ExGR	-0.04	0.35	0.15a	-0.49	0.34	-0.04b	-0.98	-0.28	-0.65c	-2.14	-1.41	-1.76d
CIVE	-69.59	-9.40	-33.37c	-58.76	24.44	-23.71c	-2.18	47.89	28.53b	70.90	104.54	88.44a
NDVI1	-0.12	0.03	-0.07d	-0.04	0.27	0.08c	0.17	0.47	0.34b	0.65	0.92	0.78a
NDVI	-0.06	0.65	0.22c	0.26	0.84	0.65b	0.55	0.96	0.81a	0.79	0.97	0.89a
RSI _546_,_1132_	0.75	0.85	0.79c	0.89	0.93	0.91b	0.86	1.02	0.92b	0.96	1.02	0.99a
RSI _534_,_750_	0.16	0.30	0.21d	0.46	0.61	0.54c	0.57	0.64	0.59b	0.67	0.82	0.72a
RSI _522_,_750_	0.14	0.26	0.19d	0.36	0.47	0.42c	0.46	0.55	0.50b	0.57	0.77	0.65a
RSI _632_,_608_	1.18	1.72	1.49a	0.95	0.99	0.97b	0.90	0.95	0.93bc	0.87	0.99	0.92c
RSI _1140_,_674_	0.23	0.48	0.31d	1.06	1.38	1.21c	1.39	1.72	1.53b	1.25	2.35	1.89a
RSI _616_,_674_	0.56	0.91	0.70c	1.24	1.31	1.28b	1.27	1.50	1.37b	1.21	1.88	1.60a
RSI _610_,_646_	0.52	0.83	0.65c	1.04	1.06	1.05b	1.09	1.17	1.12a	1.05	1.25	1.16a
RSI _622_,_640_	0.75	0.92	0.83b	1.01	1.01	1.01ab	1.02	1.08	1.05a	1.02	1.06	1.04a
RSI _648_,_1120_	1.82	2.51	2.23a	0.88	0.99	0.92b	0.72	0.78	0.75c	0.52	0.67	0.59d
RSI _620_,_618_	1.01	1.05	1.03a	1.00	1.00	1.00a	0.99	1.00	0.99ab	0.99	0.99	0.99ab
RSI _640_,_616_	1.14	1.55	1.36a	0.98	0.98	0.98b	0.89	0.98	0.93b	0.92	0.96	0.94b
RSI _584_,_650_	0.27	0.53	0.36c	0.95	1.11	1.02b	1.16	1.30	1.21a	1.03	1.46	1.27a
RSI _610_,_650_	0.51	0.83	0.65c	1.07	1.08	1.07b	1.12	1.21	1.15a	1.07	1.31	1.20a
NDVI	0.00	0.09	0.04c	0.24	0.30	0.27b	0.27	0.43	0.34ab	0.22	0.60	0.44a
NAI	-0.02	-0.01	-0.02b	-0.01	0.00	0.01ab	0.01	0.04	0.02a	-0.01	0.04	0.02a
GI	0.18	0.40	0.26d	0.94	1.30	1.10c	1.41	1.81	1.55b	1.08	2.40	1.80a

For tomato, TSS values ranged from 4.00 to 6.80 (Brix%), and firmness from 690.00 to 350.00 kg_f_/cm^2^ as listed in Table 4. While RGB indices such as INT ranged from 68.66 to 176.31, and ExR from 0.04 to 1.26 in Table 4. In addition, SRI such as, R_546_/R_1132_ values rising from 0.75 to 1.02, and NAI ranged from -0.02 to 0.04. Shravan et al. [44] reported that throughout fruit maturation, the concentration of chlorophyll decreased significantly, while the concentration of carotenoids and TSS increased. Thus the color shift that occurs during the ripening phase of fruit from green to red is due to the discovery of pre-existing pigments due to the decomposition of chlorophyll [45]. Brandt et al. [46] explained that at the beginning of the growth stage, the amount of lycopene decreases and then increases during the fruit ripening stage. Thimann [47] discovered that the chromoplasts also contain yellow or red carotenoids, such as lycopene, so we observe the breakdown or disappearance of chlorophyll with the accumulation of lycopene (a red pigment), altering the color of tomato fruit into the red.

### 3.2 Correlation analysis between all biochemical parameters of mandarin and tomato fruits

Correlation analysis has elucidated a significant association among the following variables: Chl a, Chl b, TSS, TA, TSS/TA, carotenoids (car), lycopene, and fruit firmness, as meticulously outlined in Tables 5 and 6. For mandarin fruits the greatest correlation coefficient (r) was found between Chl a, and Chl b (r = 1.00). The lowest r value was found between the TSS and TA (r = -0.75). All biochemical parameters have statistically substantial associations ranged from -0.75 to 1.00 in Table 5.

**Table 5:** Correlation coefficients among physiological parameters in mandarin fruits: chlorophyll a (Chl a), chlorophyll b (Chl b), total soluble solids (TSS), titratable acidity (TA), TSS/TA ratio, and carotenoids (Car).

	Chl a	Chl b	TSS	TA	TSS/TA	car
Chl a	1.00**					
Chl b	1.00**	1.00**				
TSS	-0.76**	-0.76**	1.00**			
TA	0.98**	0.98**	-0.75**	1.00**		
TSS/TA	-0.97**	-0.96**	0.88**	-0.97**	1.00**	
Caroten	-0.94**	-0.94**	0.85**	-0.95**	0.98**	1.00**

**. Correlation is significant at the 0.01 level (2-taile).

**Table 6:** Correlation coefficients between eight parameters, chlorophyll a (Chl a), chlorophyll b (Chl b), total soluble solids (TSS), titratable acidity (TA), TSS/TA, carotenoids (Car), lycopene, and firmness of tomato fruits.

	Chl a	Chl b	TSS	TA	TSS/TA	Firmness	car	Lycopene
Chl a	1.00**							
Chl b	0.88**	1.00**						
TSS	-0.75**	-0.78**	1.00**					
TA	-0.86**	-0.82**	0.93**	1.00**				
TSS/TA	-0.45**	-0.56**	0.87**	0.63**	1.00**			
Firmness	0.85**	0.83**	-0.88**	-0.94**	-0.60**	1.00**		
Caroten	-0.63**	-0.63**	0.80**	0.82**	0.57**	-0.84**	1.00**	
Lycopene	-0.81**	-0.77**	0.85**	0.92**	0.57**	-0.92**	0.87**	1.00**

** The correlation demonstrates statistical significance at the 0.01 significance level with a two-tailed test.

For tomato fruits, the highest r value for all biochemical parameters was between firmness and TA (r = 0.94). While the lowest correlation coefficient of—0.45 was found between Chl a and TSS/TA. All biochemical parameters of tomato have substantial significant associations ranged from -0.45 to -0.94. These results are in agreement with Elsayed et al. [9], who found that the TSS, car, Chl a, Chl b, total Chl and TA of mango were shown to be substantially linked. Galal et al. [14] discovered considerable negative relationships between TSS and Chl content of banana and orange fruit at various maturation stages.

### 3.3. Relationships between RGB indices and biochemical parameters of mandarin and tomato fruits

Twelve RGB indices acquired through digital images processing were linked to different biochemical parameters of mandarin and tomato fruits as seen in Tables 7 & 8. The findings demonstrated moderate to significant correlations between all biochemical parameters and the tested RGB indices. For mandarin fruits, IKAW and NDVI1 presented the greatest R^2^ of 0.91 with Chl a. VARI and Rn presented the highest R^2^ of 0.96 with car. The greatest R^2^ of Rn with TSS was (R^2^ = 0.81).

**Table 7:** Coefficient of determination values of linear regression models of six mandarin fruit parameters including chlorophyll b (Chl b), chlorophyll a (Chl a), total soluble solids (TSS), titratable acidity (TA), TSS/TA, carotenoids (car) with twelve RGB indices.

RGB indices	Chl a	Chl b	TSS	TA	TSS/TA	car
Rn	0.82***	0.82***	0.81***	0.84***	0.93***	0.96***
Bn	0.87***	0.87***	0.65***	0.86***	0.82***	0.80***
GRRI	0.85***	0.86***	0.77***	0.88***	0.94***	0.95***
GRVI	0.75***	0.76***	0.80***	0.79***	0.90***	0.94***
NDI	0.75***	0.76***	0.80***	0.79***	0.90***	0.94***
IKAW	0.91***	0.91***	0.67***	0.91***	0.86***	0.85***
VARI	0.80***	0.81***	0.79***	0.83***	0.93***	0.96***
ExR	0.74***	0.75***	0.80***	0.78***	0.90***	0.94***
ExGR	0.62***	0.63***	0.76***	0.67***	0.82***	0.87***
VARI1	0.82***	0.82***	0.62***	0.80***	0.77***	0.75***
NDVI1	0.91***	0.91***	0.68***	0.91***	0.86***	0.85***
NDVI	0.75***	0.76***	0.80***	0.79***	0.90***	0.94***

***Statistically significant at P ≤ 0.001.

**Table 8:** Coefficient of determination values of linear regression models of eight tomato fruit attributes, chlorophyll b (Chl b), chlorophyll a (Chl a), total soluble solids (TSS), titratable acidity (TA), TSS/TA, carotenoids (car), lycopene and firmness with twelve RGB indices.

SRIs	Chl a	Chl b	TSS	TA	TSS/TA	car	Lycopene	Firmness
Rn	0.62***	0.58***	0.75***	0.82***	0.37***	0.82***	0.86***	0.85***
GRRI	0.66***	0.59***	0.74***	0.84***	0.34***	0.76***	0.89***	0.85***
INT	0.55***	0.54***	0.73***	0.79***	0.38***	0.72***	0.76***	0.74***
GRVI	0.56***	0.53***	0.69***	0.77***	0.33***	0.83***	0.86***	0.82***
NDI	0.55***	0.53***	0.69***	0.77***	0.33***	0.83***	0.86***	0.82***
IKAW	0.66***	0.51***	0.67***	0.74***	0.36***	0.47***	0.60***	0.65***
VARI	0.57***	0.54***	0.70***	0.79***	0.33***	0.82***	0.87***	0.83***
ExR	0.56***	0.54***	0.70***	0.78***	0.34***	0.84***	0.86***	0.82***
ExGR	0.48***	0.49***	0.63***	0.70***	0.30***	0.83***	0.82***	0.77***
CIVE	0.49***	0.50***	0.62***	0.70***	0.29**	0.74***	0.83***	0.75***
NDVI1	0.66***	0.51***	0.67***	0.90***	0.36***	0.47***	0.60***	0.65***
NDVI	0.56***	0.53***	0.69***	0.79***	0.33***	0.83***	0.86***	0.82***

**,*** Statistical significance was observed at the significance levels of P ≤ 0.01 and P ≤ 0.001, respectively.

For tomato fruits, the highest R^2^ for the relationship between Chl a and GRRI, IKAW, and NDVI1 had the greatest R^2^ of 0.66. The highest R^2^ let were Chl b, with GRRI was (R^2^ = 0.59). Also, the NDVI1 produced the highest coefficient of determination (0.90) with T. acidity. The results further demonstrated that the Rn had the greatest R^2^ (0.75) with TSS, whereas ExG and Gn had the lowest R^2^ (R^2^ = 0.55). The Rn and GRRI led to the greatest R^2^ for estimating firmness (R^2^ = 0.85; for both).

In this research study, RGB indices have proven to be useful and can be used at different ripening stages of mandarin fruits. Other studies used the surface color of the fruit as one of the main factors that have been used to determine the ripeness of mandarin fruits. For instance, Fouda et al. [31] showed that the VARI index gave the highest value of R^2^ with car, Chl a and Chl b (0.85, 0.66 and 0.75, respectively. Kaur et al. [48] showed that the RGBI of the fruit images were related to the biochemical properties and a strong correlation between the average intensity of green color and fruit acidity (R^2^ = 0.99). Psiroukis et al. [49] referred to the development of a multi-scale prediction model for tomato fruit analysis. The findings of the study exhibited a substantial level of association among various VIs and the attributes of tomato plants. Notably, the (NDVI displayed a particularly strong correlation, as evidenced by a determination coefficient of 0.89 (R^2^ = 0.89). In addition, Rasool et al. [50] found that the obtained VARI and GRVI were positively associated with the Chl a content of tomato fruits (R^2^ of 0.85 and 0.86, respectively).

### 3.4. Relationships between spectral indices and biochemical parameters of mandarin and tomato fruits

All tested SRIs had significant association with the biochemical parameters with R^2^ differing from 0.16 to 0.87 for all investigated biochemical parameters of mandarin (Table 9) and tomato (Table 10). There were statistically significant associations between all assessed spectral reflectance indices derived from the visible (VIS) and near-infrared (NIR) and different biochemical properties of both fruit types. The SRIs were significantly related to all biochemical parameters of mandarin. The indices, RSI_710_,_600_, and R_730_,_650_ showed the greatest R^2^ values with respect to Chl a, Chl b with R^2^ = 0.80 for both and with T. acidity (R^2^ = 0.75), while the RSI _810_,_822_ had the greatest R^2^ with TSS (R^2^ = 0.61).

**Table 9:** Coefficient of determination values of linear regression models of six mandarin attributes, chlorophyll b (Chl b), chlorophyll a (Chl a), total soluble solids (TSS), titratable acidity (TA), TSS/TA, carotenoids (car) with twelve spectral reflectance indices.

SRIs	Chl a	Chl b	TSS	TA	TSS/TA	car
RSI _710_,_600_	0.80***	0.80***	0.42***	0.75***	0.69***	0.65***
RSI _730_,_650_	0.80***	0.80***	0.44***	0.75***	0.71***	0.67***
RSI _676_,_664_	0.56***	0.55***	0.61***	0.57***	0.66***	0.65***
RSI _672_,_670_	0.61***	0.60***	0.63***	0.61***	0.70***	0.69***
RSI _678_,_666_	0.69***	0.68***	0.59***	0.69***	0.74***	0.72***
RSI _894_/_878_	0.77***	0.76***	0.58***	0.74***	0.74***	0.69***
RSI _744_,_640_	0.78***	0.78***	0.45***	0.74***	0.70***	0.66***
RSI _826_,_640_	0.78***	0.78***	0.45***	0.74***	0.70***	0.66***
RSI _824_,_810_	0.77***	0.77***	0.57***	0.74***	0.75***	0.71***
RSI _810_,_822_	0.75***	0.75***	0.61***	0.72***	0.76***	0.73***
RSI _654_,_566_	0.56***	0.56***	0.16*	0.50***	0.39***	0.34***
NDVI	0.60***	0.59***	0.20*	0.55***	0.44***	0.39***
NAI	0.71***	0.71***	0.45***	0.68***	0.65***	0.60***
PRMI	0.72***	0.71***	0.38***	0.68***	0.62***	0.57***

*,**, *** Statistically significant at P ≤ 0.05, P ≤ 0.01, and P ≤ 0.001, respectively.

**Table 10:** Coefficient of determination values of linear regression models of eight tomato attributes, chlorophyll b (Chl b), chlorophyll a (Chl a), total soluble solids (TSS), titratable acidity (TA), TSS/TA, carotenoids (car), lycopene and firmness with spectral reflectance indices.

SRIs	Chl a	Chl b	TSS	TA	TSS/TA	car	Lycopene	Firmness
RSI _546_,_1132_	0.55***	0.44***	0.69***	0.71***	0.40***	0.45***	0.69***	0.65***
RSI _534_,_750_	0.62***	0.50***	0.70***	0.77***	0.35***	0.53***	0.81***	0.73***
RSI _522_,_750_	0.63***	0.48***	0.70***	0.76***	0.37***	0.49***	0.77***	0.70***
RSI _632_,_608_	0.50***	0.49***	0.66***	0.72***	0.33***	0.71***	0.82***	0.78***
RSI _1140_,_674_	0.67***	0.61***	0.64***	0.79***	0.25***	0.51***	0.80***	0.77***
RSI _616_,_674_	0.60***	0.59***	0.64***	0.76***	0.26***	0.60***	0.80***	0.79***
RSI _610_,_646_	0.59***	0.57***	0.70***	0.79***	0.33***	0.69***	0.87***	0.83***
RSI _622_,_640_	0.56***	0.55***	0.69***	0.76***	0.33***	0.72***	0.85***	0.82***
RSI _648_,_1120_	0.56***	0.54***	0.64***	0.74***	0.29***	0.63***	0.83***	0.80***
RSI _620_,_618_	0.53***	0.52***	0.68***	0.75***	0.33***	0.71***	0.85***	0.80***
RSI _640_,_616_	0.50***	0.51***	0.66***	0.72***	0.32***	0.72***	0.82***	0.79***
RSI _584_,_650_	0.66***	0.59***	0.70***	0.82***	0.31***	0.59***	0.86***	0.81***
RSI _610_,_650_	0.60***	0.58***	0.70***	0.80***	0.32***	0.69***	0.87***	0.83***
NDVI	0.57***	0.54***	0.53***	0.67***	0.19***	0.42***	0.65***	0.68***
NAI	0.60***	0.45***	0.46***	0.60***	0.16***	0.31**	0.55***	0.58***
GI	0.68***	0.60***	0.64***	0.79***	0.25***	0.48***	0.78***	0.75***

*,**, *** Significant statistical differences were observed at the significance levels of P ≤ 0.05, P ≤ 0.01, and P ≤ 0.001, respectively.

SRIs were found to be substantially related to tested biochemical characteristics of tomato fruits. Chl a with R^2^ values fluctuated from 0.50 to 0.68, range from 0.44 to 0.63 for Chl b, ranged from 0.46 to 0.70 for TSS, and ranged from 0.58 to 0.83 for firmness. RSI _1140_/R_674_ found to be good indicator to estimate the Chl a and Chl b, with R^2^ of 0.67 and 0.61, respectively. RSI _584_/R_65_ had well relationships with TSS and TA and the highest R^2^ values were 0.70 and 0.82, respectively. In agreement of these results, Pires et al. [51] used non-destructive assessment of the Citrus maturity based on predicting internal quality properties. This gave a good predictive performance for TSS (R^2^ = 0.79) and pH (R^2^ = 0.80) in addition to the titration acidity (R^2^ = 0.73) and maturity index (R^2^ = 0.80). Galal et al. [14] demonstrated that the NAI and R_800_/R_640_ showed the highest determination coefficient of 0.89 with chlorophyll content, and the R_570_/R_540_ index had the greatest R^2^ value (0.79) with TSS. Huang et al. [52] used spectroscopy to assess the quality of tomato and showed that the predictions of pH and TSS made by spectroscopy were high. The results showed the regression coefficient for predicting pH (R^2^ = 0.81) and the regression coefficient for TSS (R^2^ = 0.80). In addition, Wati et al. [53] found that the best-calibrated model used the wavelength range of 527–799 nm to measure the pH of intact tomato with R^2^ value of 0.90.

### 3.5. Assessing the efficacy of decision tree and random forest models in predicting quantitative attributes of mandarin and tomato produce

Table 11 delineates the implementation of Decision Tree (DT) and Random Forest (RF) models which incorporate the analysis of high-level variables using a combination of 2D-spectral vegetation indicators (VIs) and color RGB-focused indicators (RGBI). This process aids in the detection of the characteristics of mandarin maturation such as Chla, Chlb, TSS, TA, TSS/TA, and car. The DT model was trained using the 2D-VIs and RGBI to predict the examined parameters, with the reserved values of the DT model being compared against the projected values. The study’s multivariate analysis and comparison techniques indicate a significant increase in predictability when applying this approach. External verification emerged as the most dependable method for evaluating the accuracy of the regression model, as it was not employed during the model’s formulation. The results of this assessment revealed that the DT-9 SRIs model exhibited exceptional precision in the detection of Chl a, with approximately nine parameters derived from RGB data playing pivotal roles in this prediction. The corresponding R^2^ values for the training and validation datasets were 1.00, with a RMSE of 0.119 for the former and 0.99 with an RMSE of 0.080 for the latter. In the context of Chl b prediction, the DT-7RGBI model outperformed other models as the optimal predictor, demonstrating impressive R^2^ scores of 0.99 for both the training and validation datasets. The RMSE values for this model were 0.193 and 0.134 for the training and validation datasets, respectively. Turning our attention to TSS, the RF-7RGBI model emerged as the most precise model for TSS prediction, exhibiting R^2^ values of 0.96 and 0.83 for the training and validation datasets, respectively. The DT-12RGBI model was identified as the most reliable predictor for the ratio of TSS to TA, demonstrating robust R^2^ values of 0.99 for the training dataset and 0.98 for the validation dataset. The RMSE values were calculated as 0.408 for the training dataset and 0.508 for the validation dataset. These findings underscore the effectiveness of these models in predicting various biochemical parameters, thereby highlighting their potential utility in plant and soil ecology research.

**Table 11:** Results of Random Forest (RF) and Decision Tree (DT) models incorporate distinct features retrieved spectral reflectance indices (SRIs) and RGB indices (RGBI) to predict chlorophyll b (Chl b), chlorophyll a (Chl a), total soluble solids (TSS), titratable acidity (TA), TSS/TA, and carotenoids (car) of mandarin fruits.

Variable	Model	VI	Suggested features	Parameters (Md, Ms, MLn) (ntree, mtry)	Training		validation	
					R^2^	RMSE	R^2^	RMSE
Chl a	DT	SRls	PRMI, RSI_710_,_600_, RSI_810_,_822_, RSI_678_,_666_, NAI, RSI_654_,_566_, RSI_894_,_878_	(3, 2, none)	0.93***	0.462	0.75***	0.423
		RGBI	NDVI1, NDVI2, ExGR, GRVI, ExR, VARI1, VARI, rn, NDI	(3, 8, none)	1.00***	0.119	0.99***	0.080
	RF	SRls	RSI_678_,_666_, RSI_894_,_878_, RSI_654_,_566_, NAI, RSI_730_,_650_, RSI_826_,_640_, RSI_744_,_640_, RSI_676_,_664_	(2, 1)	0.783	0.820	0.75***	0.346
		RGBI	ExR, NDVI2, GRRI, VARI	(1, 11)	1.00***	0.059	0.99***	0.089
Chl b	DT	SRls	RSI_744_,_640_, RSI_824_,_810_, RSI_810_,_822_, GI, PRMI, RSI_678_,_666_, RSI_654_,_566_, RSI_894_,_878_	(5, 2, 10)	0.94***	0.621	0.77***	0.579
		RGBI	NDVI1, ExGR, ExR, VARI1, VARI, NDI, NDVI2	(3, 8, none)	0.99***	0.193	0.99***	0.134
	RF	SRls	GI, RSI_654_,_566_, NAI, RSI_710_,_600_, RSI_826_,_640_, RSI_676_,_664_	(1, 1)	0.77***	1.194	0.76***	0.541
		RGBI	GRVI, VARI, NDI, NDVI1, ExGR, rn, ExR, NDVI2	(7, 2)	1.00***	0.130	0.99***	0.138
TSS	DT	SRls	RSI_824_,_810_, RSI_678_,_666_	(3, 6, none)	0.75***	0.408	0.55***	0.434
		RGBI	ExR, ExGR, rn, GRVI, NDVI2, NDVI1, VARI, VARI1, NDI	(3, 4, none)	0.92***	0.273	0.81***	0.338
	RF	SRls	NDVI, NAI, RSI_810_,_822_, RSI_894_,_878_	(3, 5)	0.95***	0.232	0.54***	0.451
		RGBI	bn, ExR, rn, ExGR, GRVI, GRRI, VARI	(1, 7)	0.96***	0.186	0.83***	0.321
TA	DT	SRls	GI, RSI_676_,_664_, RSI_894_,_878_	(3, 4, 10)	0.91***	0.091	0.77***	0.072
		RGBI	NDVI2, GRRI	(3, 8, none)	0.98***	0.038	0.98***	0.028
	RF	SRls	RSI_894_,_878_, RSI_654_,_566_, RSI_744_,_640_, RSI_824_,_810_, PRMI	(2, 1)	0.91***	0.090	0.85***	0.067
		RGBI	GRVI, VARI, NDVI2, ExGR, NDI, ExR, GRRI	(2, 4)	0.99***	0.022	0.97***	0.026
TSS/TA	DT	SRls	NAI, RSI_894_,_878_	(3, 6, none)	0.90***	1.239	0.75***	1.234
		RGBI	Rn, GRVI, NDI, VARI1, IKAW, NDVI1, bn, VARI, ExR, NDVI2, ExGR, GRRI	(7, 2, 10)	0.99***	0.408	0.98***	0.508
	RF	SRls	NDVI, RSI_672_,_670_, RSI_678_,_666_, RSI_676_,_664_, RSI_654_,_566_, GI, RSI_894_,_878_, RSI_710_,_600_, RSI_730_,_650_, RSI_824_,_810_	(2, 1)	0.81***	1.737	0.69***	1.275
		RGBI	GRVI, ExGR, bn, NDI, ExR, VARI	(1, 3)	0.99***	0.402	0.97***	0.629
Car	DT	SRls	GI, RSI_894_,_878_	(5, 2, none)	0.95***	0.011	0.77***	0.015
		RGBI	ExR, VARI	(3, 2, none)	0.98***	0.008	0.96***	0.008
	RF	SRls	RSI_894_,_878_, RSI_824_,_810_, RSI_672_,_670_, RSI_676_,_664_, RSI_678_,_666_, GI, PRMI, RSI_710_,_600_, RSI_826_,_640_, RSI_810_,_822_, RSI_744_,_640_, RSI_654_,_566_, NAI, NDVI, RSI_730_,_650_	(1, 6)	0.94***	0.012	0.71***	0.019
		RGBI	NDVI1, NDVI2, bn, GRVI, ExGR, NDI, rn, ExR, GRRI	(4, 17)	1.00***	0.004	0.97***	0.008

Where: Md represents the maximum depth of the tree, Ms corresponds to the minimum samples per leaf, and Mln denotes the maximum number of leaf nodes.

*** Statistically significant at P ≤ 0.001.

The major variables of tomato fruits were isolated through the parameters researched, as depicted in Table 12, leveraging both DT and RF models. These particular features were instrumental in pinpointing Chl a, Chl b, TSS, TA, TSS/TA, Hardness, Carotene, and Lycopene. The efficacy and precision (accuracy and RMSE) of DT and RF models in predicting the considered parameters are represented in Table 12. The study reveals that the model DT-3 SRIs maintained the supreme correlation with Chl a and distinctive features, making it the optimum model for predictions. It encompasses approximately three critical attributes significant for predicting Chl a. The resultant R^2^ values were 0.87 (RMSE = 0.091) and 0.740 (RMSE = 0.087) for training and verification sets, respectively. For assessing Chl b, the RF-22 SRIs demonstrated superior results, achieving an R^2^ of 0.84 (RMSE = 0.099) and 0.58 (RMSE = 0.125) in the training and validation series, correspondingly. The DT-2RGBI, which was the most precise for TSS determination, had RMSE records of 0.248 and 0.224 and R^2^ statistics of 0.83 and 0.76 for the training and validation series, respectively. The DT-7VIs model predicted the TSS/TA ratio accurately, with R^2^ values of 0.62 and 0.39 (RMSE = 0.231 and 0.222) for training and validation sets, respectively. The RF-9RGBI model surpassed in predicting hardness, concluding with R^2^ values of 0.96 and 0.87 (RMSE = 21.039 and 25.194) for the training and validation, respectively. The DT-2RGBI model precisely predicted carotene, with R^2^ values of 0.89 and 0.86 (RMSE = 0.014 and 0.011) for both series. Based on the outcomes from [54, 55], enhancing the regression methods for accurate forecasting required strategies such as adjusting high-level features and modifying model settings.

**Table 12:** Shows the results of Random Forest (RF) and Decision Tree (DT) models incorporate distinct features retrieved spectral reflectance indices (SRIs) and RGB indices (RGBI) to predict chlorophyll b (Chl b), chlorophyll a (Chl a), total soluble solids (TSS), titratable acidity (TA), TSS/TA, carotenoids (car), lycopene and firmness of tomato fruits.

Variable	Model	VI	Suggested features	Parameters (Md, Ms, MLn) (ntree, mtry)	Training		Validation	
					R^2^	RMSE	R^2^	RMSE
Chl a	DT	SRls	RSI_534_,_750_, RSI_610_,_646_, RSI_648_,_1120_	(3, 2, none)	0.87***	0.091	0.74***	0.087
		RGBI	NDVI2, ExR, NDVI1, NDI, rn	(3, 10, none)	0.78***	0.116	0.70***	0.092
	RF	SRls	RSI_648_,_1120_	(1, 3)	0.84***	0.099	0.68***	0.088
		RGBI	VARI, NDVI1, ExR, GRVI, IKAW, CIVE, NDVI2, rn	(7, 5)	0.90***	0.078	0.70***	0.098
Chl b	DT	SRls	RSI_640_,_616_, PRMI, RSI_1120_,_674_, RSI_6481120_, RSI_616_,_674_	(3, 8, 10)	0.75***	0.126	0.51***	0.129
		RGBI	rn, VARI	(3, 10, none)	0.67***	0.145	0.51***	0.137
	RF	SRls	RSI_620_,_618_, RSI_626_,_600_, GI, RSI_610_,_646_, RSI_622_,_640_, RSI_522_,_750_, RSI_1140_,_674_, NDVI, RSI_534_,_750_, NAI, RSI_546_,_1132_, RSI _716_,_744_, RSI_640_,_616_, RSI_744_,_710_, PRMI, RSI_504_,_704_, RSI_1120_,_674_, RSI_584_,_650_, RSI_632_,_608_, RSI_616_,_674_, RSI_648_,_1120_, RSI_610_,_650_	(8, 5)	0.84***	0.099	0.58***	0.125
		RGBI	CIVE, NDI, rn, ExGR, IKAW	(1, 7)	0.90***	0.079	0.54***	0.133
TSS	DT	SRls	RSI_504_,_704_, RSI_1140_,_674_, RSI_640_,_616_	(3, 4, 10)	0.86***	0.224	0.74***	0.243
		RGBI	NDVI2, rn	(3, 10, none)	0.83***	0.248	0.76***	0.224
	RF	SRls	RSI_1140,674_, RSI_610,646_, RSI_546,1132_, RSI_534,750_, RSI _522,750_, RSI_632,608_, RSI_504,704_, RSI_616,674_, RSI_622,640_	(1, 15)	0.94***	0.140	0.71***	0.254
		RGBI	INT, rn	(1, 15)	0.96***	0.120	0.73***	0.227
TA	DT	SRls	RSI_648_,_1120_, RSI_622_,_640_	(3, 8, none)	0.91***	0.016	0.84***	0.014
		RGBI	IKAW, ExR, NDVI2, NDVI1, INT, rn	(3, 6, none)	0.95***	0.012	0.89***	0.010
	RF	SRls	RSI_1120,674_, RSI_648,1120_, RSI_610,646_, RSI_610,650_, RSI_504,704,_ NDVI, RSI_744,710_, RSI_622,640_, RSI_584,650_, GI, RSI_1140,674_, RSI_626,600_, RSI_640,616_, RSI_534,750_, RSI_620,618_	(2, 4)	0.95***	0.012	0.80***	0.014
		RGBI	NDVI1, rn	(1, 2)	0.95***	0.012	0.85***	0.012
TSS/TA	DT	SRls	RSI_640_,_616_, RSI_610_,_650_, RSI _744_,_710_, NDVI, RSI_716_,_744_, RSI _504_,_704_, RSI_622_,_640_	(5, 8, none)	0.62***	0.231	0.39***	0.222
		RGBI	ExGR, NDVI2, IKAW, VARI, INT	(3, 10, none)	0.52***	0.259	0.27***	0.231
	RF	SRls	NAI, RSI_546,1132_, RSI _616,674_, RSI_534,750_, RSI_504,704_, RSI _522,750_	(1, 10)	0.84***	0.151	0.33***	0.234
		RGBI	GRRI, ExGR, IKAW, VARI, NDVI2, rn, INT	(7, 14)	0.86***	0.142	0.21**	0.239
Car	DT	SRls	RSI_534_,_750_, RSI_622_,_640_	(7, 2, none)	0.93***	0.011	0.70***	0.015
		RGBI	IKAW, rn	(5, 10, none)	0.89***	0.014	0.86***	0.011
	RF	SRls	RSI_584,650_, RSI_112,674_, NDVI, RSI_546,1132_, PRMI, RSI_504,704_, NAI, RSI_534,750_, RSI_522,750_, RSI_616,674_, RSI_632,608_, RSI_610,650_, RSI_640,616_, RSI_622,640_	(8, 13)	0.92***	0.012	0.70***	0.014
		RGBI	NDVI1, NDI, CIVE, rn	(1, 14)	0.97***	0.007	0.84***	0.013
Lycopene	DT	SRls	RSI_632,608_, RSI_648,1120_	(3, 4, 10)	0.95***	4.588	0.89***	4.442
		RGBI	ExGR, NDVI2, NDI, IKAW, NDVI1, CIVE, rn	(3, 8, none)	0.95***	4.633	0.91***	4.424
	RF	SRls	RSI_546,1132_, RSI_626,600_, NAI, RSI_640,616_, RSI_632,608_, RSI_1120,674_, RSI_620,618_, RSI_504,704_, GI, RSI_610,646_, RSI_648,1120_, RSI_522,750_, RSI_1140,674_, RSI_622,640_, RSI_610,650_, RSI_584,650_	(3, 11)	0.973***	3.300	0.88***	4.474
		RGBI	VARI, ExR, ExGR, NDI, NDVI2, NDVI1, CIVE, GRVI, rn, GRRI	(9, 2)	0.939***	4.938	0.879***	4.888
Firmness	DT	SRls	RSI_632,608_, RSI_640,616_	(3, 10, none)	0.88***	34.274	0.84***	29.152
		RGBI	NDI, IKAW, ExR, CIVE, NDVI1, NDVI2, VARI, rn, GRVI, ExGR	(3, 10, none)	0.92***	27.808	0.87***	26.867
	RF	SRls	RSI_504,704_, RSI_546,1132_, NDVI, RSI_534,750_, NAI, RSI_716,744_, RSI_744,710_, RSI_584,650_, RSI_1120,674_, RSI_522,750_, RSI_648,1120_, RSI_610,646_, RSI_610,650_, RSI_626,600_, RSI_640,616_, RSI_620,618_, RSI_622,640_, RSI_632,608_	(4, 15)	0.96***	19.829	0.83***	32.075
		RGBI	GRVI, VARI, IKAW, NDVI2, NDI, ExGR, NDVI1, GRRI, ExR	(5, 5)	0.96***	21.039	0.87***	25.194

The SRIs and RGBIs were processed using two ML models, the DT and RF, with the goal of selecting optimal features in each iteration. Subsequently, the most effective hybrid variables (HV) were identified to estimate the quality of different fruits, including mandarins (Table 13) and tomatoes (Table 14). First, regarding mandarin fruits: In the task of predicting Chl a, the DT-2HV model delivered exceptional results, registering an R^2^ of 0.993 with an RMSE of 0.149 for the training set, and an R^2^ of 0.991 with an RMSE of 0.114 for the validation set. For the prediction of Chl b, the RF-23HV model demonstrated excellent accuracy, with R^2^ values of 0.996 and 0.989 and RMSE values of 0.146 and 0.166 for the training and verification sets, respectively. Exceptional accuracy was shown by the RF-14HV model in predicting TSS, as evidenced by R^2^ values of 0.819 for the training set and 0.688 for the verification set, and corresponding RMSEs of 0.348 and 0.337. In predicting TA, the RF-16HV model stood out, securing an R^2^ of 0.994 and RMSE of 0.023 for the training set, alongside an R^2^ of 0.984 and RMSE of 0.026 for the verification set. With outstanding precision, the RF-12HV model predicted TSS/TA, recording R^2^ scores of 0.993 (RMSE = 0.325 and 0.979 (RMSE = 0.448) for the training and verification sets, respectively. In the evaluation of car prediction, the DT-2HV model showcased remarkable results, with an R^2^ of 0.988 and an RMSE of 0.005 for training, and an R^2^ of 0.977 with an RMSE of 0.006 for verification.

**Table 13:** Displays the results from the Random Forest (RF) and Decision Tree (DT) models, using the superior hybrid features of both spectral reflectance indices (SRIs) and RGB indices (RGBIs) together to predict various characteristics of mandarin fruits. These characteristics include chlorophyll b (Chl b), chlorophyll a (Chl a), total soluble solids (TSS), titratable acidity (TA), the TSS/TA ratio, and carotenoids (car).

Variable	Model	Advanced characteristics of integrating SRIs and RGBIs	Parameters (Md, Ms, MLn) (ntree, mtry)	Training		Cross-validation	
				R^2^	RMSE	R^2^	RMSE
Chl a	DT	ExGR, NDI	**(3, 8, None)**	**0.993**	**0.149**	**0.991**	**0.114**
	RF	NDI, NDVI2	(1, 15)	0.998	0.065	0.990	0.117
Chl b	DT	rn, GRVI	(3, 8, None)	0.990	0.242	0.988	0.175
	RF	RSI_730,650_, VARI1, RSI_824,810_, RSI_678,666_, RSI_744,640_, RSI_894,878_, VARI, RSI_710,600_, rn, ExGR, RSI_826,640_, GI, GRVI, RSI_676,664_, NDVI, NAI, RSI_810,822_, RSI_654,566_, RSI_672,670_, PRMI, NDVI2, GRRI, ExR	**(1, 5)**	**0.996**	**0.146**	**0.989**	**0.166**
TSS	DT	NAI, RSI_744,640_, RSI_894,878_, RSI_810,822_, rn	(3, 8, None)	0.785	0.379	0.722	0.359
	RF	GRVI, bn, RSI_654,566_, IKAW, RSI_672,670_, rn, RSI_824,810_, VARI1, RSI_744,640_, ExGR, RSI_810,822_, NAI, NDI, VARI	**(4, 2)**	**0.819**	**0.348**	**0.688**	**0.337**
TA	DT	RSI_824,810_, RSI_654,566_, NAI, NDVI1, PRMI, rn, ExGR, VARI	(3, 8, None)	0.977	0.045	0.973	0.029
	RF	RSI_826,640_, RSI_672,670_, NDI, RSI_654,566_, ExGR, RSI_676,664_, bn, NDVI1, NDVI2, GRRI, RSI_744,640_, PRMI, VARI, ExR, RSI_824,810_, GRVI	**(10, 1)**	**0.994**	**0.023**	**0.984**	**0.026**
TSS/TA	DT	RSI_710,600_, VARI1, ExR, VARI, IKAW, NDI, GRVI, GRRI, bn, rn, PRMI, NDVI1, GI, NDVI, RSI_654,566_, RSI_810,822_, RSI_824,810_, RSI_826,640_, RSI_744,640_, RSI_894,878_, RSI_678,666_, RSI_672,670_, RSI_676,664_, RSI_730,650_, NAI, ExGR, NDVI2	(3, 8, None)	0.983	0.523	0.969	0.522
	RF	rn, RSI_730,650_, RSI_654,566_, RSI_894,878_, ExGR, RSI_678,666_, NDVI, NDVI1, RSI_824,810_, VARI, ExR, GRVI	**(10, 2)**	**0.993**	**0.325**	**0.979**	**0.448**
Car	DT	RSI_894,878_, VARI	**(3, 4, None)**	**0.988**	**0.005**	**0.977**	**0.006**
	RF	VARI1, RSI_826,640_, NDVI2, GRRI, ExGR, RSI_824,810_, RSI_678,666_, RSI_894,878_, bn, GI, rn, RSI_744,640_, RSI_810,822_, IKAW, GRVI, NAI, RSI_676,664_, PRMI, NDVI1, NDVI, ExR, RSI_730,650_, VARI, NDI	(2, 3)	0.987	0.006	0.969	0.006

**Table 14:** Presents the results of the Random Forest (RF) and Decision Tree (DT) models, which utilize advanced hybrid features from both spectral reflectance indices (SRIs) and RGB indices (RGBIs) together to predict various properties of tomato fruits. These properties include chlorophyll b (Chl b), chlorophyll a (Chl a), total soluble solids (TSS), titratable acidity (TA), the TSS/TA ratio, carotenoids (Car), lycopene, and firmness.

Variable	Model	Advanced characteristics of integrating SRIs and RGBIs	Parameters (Md, Ms, MLn) (ntree, mtry)	Training		Cross-validation	
				R^2^	RMSE	R^2^	RMSE
Chl a	DT	NDVI2, RSI_504_,_704_, RSI_648_,_1120_, RSI_1120_,_674_, rn	**(3, 2, None)**	**0.905**	**0.077**	**0.785**	**0.077**
	RF	RSI_610,650_, RSI_648,1120_, RSI_1140,674_, NDVI2, RSI_610,646_, rn	(1, 11)	0.943	0.059	0.775	0.084
Chl b	DT	RSI_1120_,_674_, NDVI2, RSI_648_,_1120_, RSI_616_,_674_	**(3, 6, None)**	**0.779**	**0.119**	**0.574**	**0.116**
	RF	RSI_610,646_, NDVI2, RSI_584,650_, RSI_716,744_, RSI_648,1120_, rn, IKAW, RSI_1120,674,_ RSI_616,674_, CIVE	(4, 8)	0.887	0.085	0.583	0.125
TSS	DT	GRRI, IKAW, RSI_584,650_, rn, RSI_640,616_	(3, 10, None)	0.831	0.244	0.773	0.212
	RF	RSI_648,1120_, RSI_716,744_, GI, NDVI1, RSI_640,616_, PRMI, NDVI, NAI, RSI_744,710_, VARI, RSI_522,750_, CIVE, IKAW, RSI_584,650_, ExGR, RSI_1140,674_, INT, NDVI2, RSI_632,608_, RSI_616,674_, RSI_610,650_, GRVI, ExR, RSI_622,640_, RSI_546,1132_, rn, RSI_504,704_	**(16, 13)**	**0.960**	**0.118**	**0.793**	**0.207**
TA	DT	NDVI2, ExR, VARI, IKAW, NDI, GRVI, RSI_584,650_, GRRI, PRMI, GI, NAI, NDVI, RSI_610,650_, NDVI1, INT, RSI_504/704_, RSI_620,618_, RSI_522,750_, RSI_632,608_, RSI_626,600_, RSI_1120,674_, RSI_1140,674_, RSI_640,616_, RSI_534,750_, RSI_610,646_, RSI_744,710_, RSI_716,744_, RSI_648,1120_, RSI_616,674_, CIVE, RSI_622,640_, rn	(3, 10, None)	0.935	0.014	0.876	0.010
	RF	RSI_504,704_, RSI_546,1132_, RSI_1120,674_, RSI_648,1120_, RSI_744,710_, RSI_1140,674_, VARI, GRRI, IKAW, CIVE, INT, GRVI, RSI_632,608_, RSI_522,750_, RSI_622,640_, RSI_534,750_, NDI, ExR, RSI_640,616_, rn	**(4, 11)**	**0.982**	**0.007**	**0.896**	**0.010**
TSS/TA	DT	RSI_616,674_ RSI_504,704_, INT	(1, 2, None)	0.372	0.295	0.284	0.233
	RF	RSI_616,674_, NAI, INT, NDVI2, RSI_522,750_, RSI_546,1132_, RSI_504,704_	**(3, 11)**	**0.5515**	**0.176**	**0.364**	**0.218**
Car	DT	NDVI2, CIVE	**(5, 10, None)**	**0.891**	**0.013**	**0.873**	**0.011**
	RF	GI, RSI_744,710_, NAI, RSI_622,640_, RSI_626,600_, GRRI, RSI_1140,674_, NDVI2, IKAW, RSI_534,750_, NDVI1, RSI_616,674_, ExR, rn, CIVE, NDI	(8, 15)	0.970	0.007	0.852	0.012
Lycopene	DT	RSI_610,650_, VARI, CIVE, RSI_648,1120_	**(5, 4, 10)**	**0.978**	**2.969**	**0.919**	**3.618**
	RF	CIVE, NDVI, RSI_640,616_, RSI_620,618_, IKAW, RSI_648,1120_, INT, NDVI2, rn, RSI_1120,674_, RSI_744,710_, RSI_504,704_, ExGR, RSI_626,600_, ExR, RSI_584,650_	(11, 5)	0.974	3.254	0.935	3.679
Firmness	DT	RSI_640,616_, RSI_504,704_, RSI_744,710_, RSI_716,744_, RSI_648,1120_, rn, ExGR, RSI_622,640_	(3, 8, None)	0.937	24.762	0.894	24.891
	RF	RSI_616,674_, NDVI2, INT, RSI_716,744_, IKAW, VARI, rn, ExGR, NDVI1, RSI_640,616_, RSI_622,640_, RSI_610,646_	**(3, 8)**	**0.973**	**16.251**	**0.898**	**23.199**

Second, for tomato fruits: the DT-5HV model demonstrated exemplary performance in the Chl a prediction, achieving an R^2^ of 0.905 and an RMSE of 0.077 for the training dataset, and an R^2^ of 0.785 with an RMSE of 0.077 for the validation dataset. For Chl b assessments, the DT-4HV model proved exceptionally precise, attaining R^2^ records of 0.779 and 0.574 and RMSE measurements of 0.119 and 0.116 for the training and validation datasets, respectively. Precision was evident in the DT-27HV model’s TSS predictions, which showed an R^2^ of 0.960 (RMSE = 0.118) for training and 0.793 (RMSE = 0.207) for validation, respectively. The DT-20HV model’s performance in TA prediction was notably strong, with an R^2^ of 0.982 and RMSE of 0.007 during training, and an R^2^ of 0.896 and RMSE of 0.010 during validation. The RF-7HV model attained moderate accuracy in estimating TSS/TA, with an R^2^ of 0.5515 and RMSE = 0.176 during training, and an R^2^ of 0.364 and RMSE = 0.218 in evaluation. The DT-2HV model’s forecasting of the Car was robust, recording R^2^ scores of 0.891 and 0.873 and RMSEs of 0.013 and 0.011 for the training and validation phases, respectively. The DT-4HV model outperformed in lycopene prediction, registering an R^2^ of 0.978 and an RMSE of 2.969 in training, followed by an R^2^ of 0.919 and an RMSE of 3.618 in validation. In firmness calculation, the DT-12HV model achieved exceptionally, securing an R^2^ of 0.973 with an RMSE of 16.251 during the training phase, and capturing an R^2^ of 0.898 with an RMSE of 23.199 in the validation phase. These findings are consistent with previous studies [8, 53], which indicate that selecting high-level variables plays a crucial role in enhancing model optimization and improving the accuracy of the predicted outcomes.

In the future, leveraging advanced ML techniques with optimally combined features holds significant promise for further enhancing the accuracy and efficiency of fruit quality assessment. Continued refinement of these combined features and the exploration of novel algorithms can lead to more robust predictive models, applicable to a broader range of fruits and agricultural products. Additionally, integrating larger datasets and incorporating real-time data processing capabilities will facilitate more dynamic and precise quality monitoring systems, ultimately benefiting producers and consumers alike through improved product consistency and reduced waste.

## 4. Conclusions

A cost-effective approach to appraise the fruit quality parameters of mandarin and tomato at different levels of ripeness was developed using RGB and SRI indices in combination with the DT and RF models. In the context of R^2^ values, the RGBI and newly created SRIs outperformed to assess the fruit quality parameters of mandarin and tomato. All tested RGB and SRIs examined had significant association with the biochemical parameters of mandarin and tomato. There are statistically significant associations between all assessed SRIs derived from the VIS and NIR regions and fruit attributes. Combining RGB and SRIs indices with DT and RF models would be a robust strategy for estimating eight observed variables associated with reasonable accuracy. The findings of this research study would be adequate to provide a potential reference for estimating several parameters. This research also offers technological assistance for monitoring and assessing the fruit quality of mandarin and tomato during ripening and storage. In conclusion, this information, which is based upon the most reliable RGB, SRI, and DT and RF calibration models developed in the current study, has the potential to be used to build active measurement systems in order to track fruit quality and ripeness in the field or factory.

## Supporting information

S1 Dataset (RAR)
